# Proteomic analysis unveils Gb3-independent alterations and mitochondrial dysfunction in a *gla*^*−/−*^ zebrafish model of Fabry disease

**DOI:** 10.1186/s12967-023-04475-y

**Published:** 2023-09-05

**Authors:** Hassan Osman Alhassan Elsaid, Mariell Rivedal, Eleni Skandalou, Einar Svarstad, Camilla Tøndel, Even Birkeland, Øystein Eikrem, Janka Babickova, Hans-Peter Marti, Jessica Furriol

**Affiliations:** 1 Department of Clinical Medicine, University of Bergen, Bergen, Norway; 2 Department of Medicine, Haukeland University Hospital, Bergen, Norway; 3 Department of Pediatrics, Haukeland University Hospital, Bergen, Norway; 4 Institute of Molecular Biomedicine, Faculty of Medicine, Comenius University, Bratislava, Slovakia

**Keywords:** Lysosome, Mitochondria, Stress, Fabry disease

## Abstract

**Background:**

Fabry disease (FD) is a rare lysosomal storage disorder caused by mutations in the GLA gene, resulting in reduced or lack of α-galactosidase A activity. This results in the accumulation of globotriaosylceramide (Gb3) and other glycosphingolipids in lysosomes causing cellular impairment and organ failures. While current therapies focus on reversing Gb3 accumulation, they do not address the altered cellular signaling in FD. Therefore, this study aims to explore Gb3-independent mechanisms of kidney damage in Fabry disease and identify potential biomarkers.

**Methods:**

To investigate these mechanisms, we utilized a zebrafish (ZF) *gla*^*−/−*^ mutant (MU) model. ZF naturally lack A4GALT gene and, therefore, cannot synthesize Gb3. We obtained kidney samples from both wild-type (WT) (n = 8) and MU (n = 8) ZF and conducted proteome profiling using untargeted mass spectrometry. Additionally, we examined mitochondria morphology and cristae morphology using electron microscopy. To assess oxidative stress, we measured total antioxidant activity. Finally, immunohistochemistry was conducted on kidney samples to validate specific proteins.

**Results:**

Our proteomics analysis of renal tissues from zebrafish revealed downregulation of lysosome and mitochondrial-related proteins in *gla*^*−/−*^ MU renal tissues, while energy-related pathways including carbon, glycolysis, and galactose metabolisms were disturbed. Moreover, we observed abnormal mitochondrial shape, disrupted cristae morphology, altered mitochondrial volume and lower antioxidant activity in *gla*^*−/−*^ MU ZF.

**Conclusions:**

These results suggest that the alterations observed at the proteome and mitochondrial level closely resemble well-known GLA mutation-related alterations in humans. Importantly, they also unveil novel Gb3-independent pathogenic mechanisms in Fabry disease. Understanding these mechanisms could potentially lead to the development of innovative drug screening approaches. Furthermore, the findings pave the way for identifying new clinical targets, offering new avenues for therapeutic interventions in Fabry disease. The zebrafish *gla*^*−*/−^ mutant model proves valuable in elucidating these mechanisms and may contribute significantly to advancing our knowledge of this disorder.

**Graphical Abstract:**

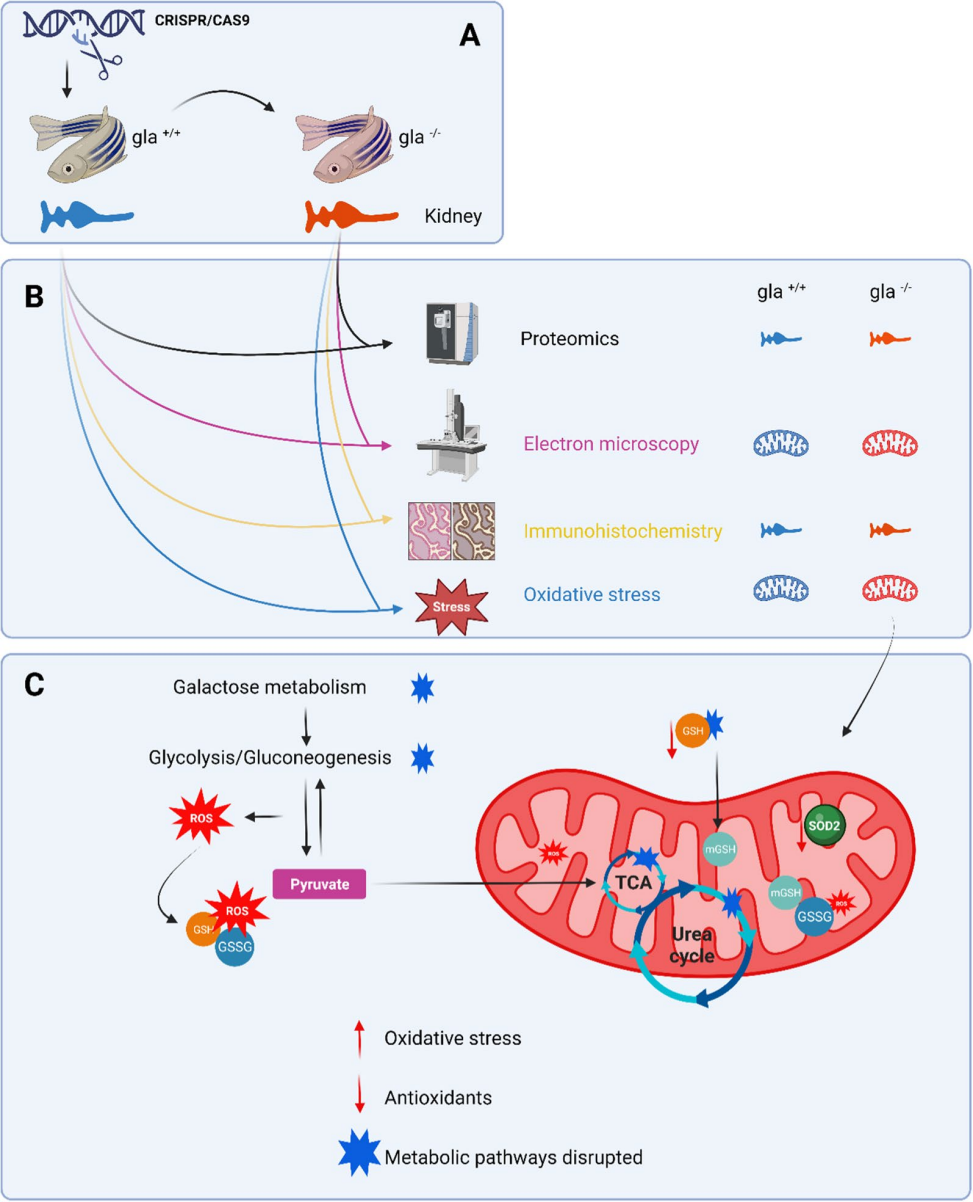

**Supplementary Information:**

The online version contains supplementary material available at 10.1186/s12967-023-04475-y.

## Background

Fabry disease (FD, OMIM #301500) is a rare genetic lysosomal storage disorder due to partial or complete deficiency of lysosomal α-galactosidase A (α-GAL; EC 3.2.1.22) activity [1, 2]. Since this enzyme hydrolyzes polysaccharides, glycolipids, and glycopeptides by cleaving α-galactose residues, its deficiency results in a multi-systemic, progressive lysosomal accumulation of globotriaosylceramide (Gb3), globotriaosylsphingosine (lysoGb3), and other related glycosphingolipids such as galabiosylceramide Ga2, particularly affecting endothelial, renal, cardiac, and nerve cells. Progressive deposition of these glycosphingolipids leads to significant cellular impairments and organ failures [3].

A gradual accumulation of Gb3 and lysoGb3 is most frequently used as a marker for both diagnosis and monitoring of FD, and current therapies focus on reducing the Gb3 load by enzyme replacement therapy (ERT), chaperone, or gene therapy [4, 5]. Although these treatments are able to decrease Gb3 deposits [6–8], their effectiveness and impact on tissue damage is still unclear [9, 10]. Numerous mutations are described leading to an increasing understanding of the heterogeneity and complexity of the disease, as well as recent insight in limitations of current therapy [11].

Although Fabry disease is a multisystemic pathology, kidneys are one of the primary organs affected [12]. In Fabry patients, Gb3 accumulates in virtually all kidney cell types [13]. This accumulation can lead to a decline in kidney function over time, which can eventually progress to end-stage renal disease (ESRD) [14]. Thus, studying the kidneys in Fabry disease is important to understand the underlying mechanisms of the disease and to develop effective treatment strategies that can slow or prevent kidney damage. Additionally, monitoring kidney function is an essential part of managing the disease, as kidney damage is one of the most severe and debilitating complications of Fabry disease [15]. Omics-related technologies are increasingly being used to enhance understanding of FD biology, and to reveal additional biomarkers hopefully leading to earlier diagnosis and more timely interventions [16]. Zebrafish (ZF) is a widely used model organism for human diseases [17], and its potential in the study of lysosomal storage diseases, i.e. Gaucher disease, has repeatedly been highlighted [18, 19]. Recently, we have demonstrated that ZF can also be advantageously used in the study of FD [20].

In FD, it has been increasingly recognized that current therapy only partly benefits the clinical course and may mask other FD biomarkers of potential clinical relevance. One main challenge is to distinguish between Gb3-dependent and Gb3-independent renal damage [11]. Indeed, Gb3 and lysoGb3 are not the only α-GAL substrates and other molecules and molecular pathways are suspected to impact disease development [10, 21, 22]. One mechanism proposed is based on unfolded protein response (UPR). Recent studies in FD have shown that UPR can generate endoplasmic reticulum stress, mitochondrial stress and lysosomal stress prior to the Gb3 accumulation [23, 24]. Briefly, in normal cellular state, the UPR is not active, however, in the case of accumulation of unfolded proteins, UPR is initiated to reduce the load of unfolded proteins and minimize damage due to stress [25]. Since ZF genome lacks a4galt, the gene encoding for the enzyme responsible for Gb3 synthesis [26, 27], this model is of particularly interesting in FD research. Indeed, we have shown that this model can mirror major FD nephropathy characters in the absence of Gb3 accumulation [20]. Importantly, to our knowledge, this is the first study that explores proteome differences in a Gb3-free model of FD.

Using a the *gla*^−/−^ mutant ZF model, this study aims to unravel FD mechanisms associated with kidney damage related to α-Gal deficiency in the absence of Gb3 accumulation and identify novel markers of potential clinical relevance.

## Methods

### Ethical approval

The Norwegian Food Safety Authority (Mattilsynet) approved the study (FOTS ID 15256). All procedures were performed following the Zebrafish Facility guidelines of the University of Bergen. These guidelines do not require permission for testing ZF embryos before the free-feeding stage (5 dpf). Instead, following the ZF facility guidelines, all invasive pain-causing interventions on stages older than five dpf were performed under anesthetic conditions. All zebrafish used in this study were randomized, and the researchers were not blinded when conducting experiments.

### Zebrafish maintenance

The AB/Tübingen (AB/TU) ZF strain was used in all experiments. Eggs, embryos, larvae, juveniles, and adult fish were handled in compliance with applicable national and international standards, according to ZF facility regulations at the University of Bergen. Under normal laboratory conditions, adult (90 + days post-fertilization dpf) ZF were held at 28 °C on a 14 h light/10 h dark period. Standard spawning protocol (www.zfin.org), was followed by egg harvesting. Eggs were stored in an E3 medium containing 0.01% methylene blue after harvesting. Embryos and larvae were incubated at 28 °C until 5 dpf.

### Generation of *gla*^*−/−*^ model in zebrafish

A more detailed model generation is described in [20]. Briefly, Zebrafish *gla* mutants were generated using CRISPR/Cas9-mediated gene targeting. One-target region, located within *gla* exon five was chosen for sgRNA recognition. The corresponding sgRNA was injected into wild-type zebrafish embryos (N = 200) at the 1-cell stage together with cas9 protein. For mutation screening, sgRNA-injected embryos (Founder 0/F0) of 5 dpf were screened by PCR fragment analysis to confirm successful mutant generation. After the second generation (F2), individuals were genotyped and homozygous *gla*^*−/*−^ MU and *gla*^+*/*+^ WT were selected for further analysis. In *gla*^*−/*−^, the mutation generated frameshift in the mRNA as was indicated by the sequencing analysis. However, the enzyme activity decreased by approximately 70% compared to the wild-type, with a significant difference between the wild-type and mutant (p-value = 0.004).

### Sample collection and protein extraction

Whole kidneys were collected from adult, 6-month-old fish (N = 32, 16/genotype) and samples were pooled (2/sample, male N = 8, female N = 8). Briefly, ZF were euthanized in 300 mg/L tricaine methane sulfonate MS222 (Sigma, Cat. No. A-5040) and dissected. Kidneys were exposed after discarding the viscera under cold 1X PBS (Life Technologies, Cat. No. AM9625). Removed organs were washed in 1X PBS and snap-frozen in liquid nitrogen immediately after collection.

For protein extraction, 30 μL of RIPA lysis buffer (ThermoFisher, Cat. No. 89901) with protease inhibitor cocktail (Roche, Cat. No. 04693124001) and phosphatase inhibitor cocktail (Sigma‐Aldrich, Cat. No. P5726) was added to each sample and tissues were disintegrated using the Precellys Evolution Homogenizer and soft tissue homogenizing kit CK14 (both from Bertin Instruments). BCA protein assay (ThermoFisher, Cat. No. 23235) was performed to measure protein concentrations.

For every 20 μg of proteins, 3 μL of 100mM DTT were added to reduce samples (20 min at 60 °C) and 4 μL of 200 mM iodoacetic acid (IAA) for cysteine alkylation (1 h at room temperature). For protein cleanup and digestion, we added 200 μg of prepared SP3 beads (Automated Magnetic Separations for Proteomics, ThermoFisher) and 100% ethanol to a final concentration of 70% to induce protein binding and incubated the mixture at 24 °C (RT) for 7 min. Afterward, tubes were placed in a magnetic rack and incubated until the beads migrated to the tube wall. Unbound supernatant was discarded. 180 μL of 80% ethanol SP3 rinse solution was used to reconstitute and rinse (3X) the beads. Finally, 50 μL/sample of 0.8 μg of trypsin in 100 mM AmBic/1 mM CaCl_2_ solution was added for peptide digestion, and tubes were sonicated for 30 s in a water bath to fully disaggregate the beads, and then incubated at 37 °C for 16 h. Next, tubes were centrifuged at 13,000 rpm at 24 °C for 3 min. Tubes were then rested on a magnetic rack until the beads had settled onto the tube wall. The supernatant was then passed into a new tube. Peptides were further diluted with 50 µl of 0.5M NaCl. Finally, peptides were desalted using Oasis C18 30 µg Elution plates (Waters, Milford, MA), and dried in a vacuum centrifuge.

Dried peptide mixtures were resuspended in 200 mM HEPES, pH 8. Tandem Mass Tag (TMT) 16plex label reagent set was used for the identification and quantification of proteins following the manufacturer’s protocol (cat. # A44520, ThermoFisher). After labeling and pooling, samples were desalted using Oasis C18 30 µg Elution plates (Waters, Milford, MA), and dried in a vacuum centrifuge.

### NanoLC-ESI- Orbitrap exploris mass spectrometry

About 0.5 μg protein as tryptic peptides dissolved in 2% acetonitrile (ACN), and 0.5% formic acid (FA), were injected into an Ultimate 3000 RSLC system (Thermo Scientific, Sunnyvale, California, USA) connected online to an Orbitrap Exploris mass spectrometer (Thermo Scientific, San Jose, CA, USA) equipped with EASY-spray nano-electrospray ion source (Thermo Scientific). The sample was loaded and desalted on a pre-column (Acclaim PepMap 100, 2 cm × 75 µm ID nanoViper column, packed with 3 µm C18 beads) at a flow rate of 5 µl/min for 5 min with 0.1% TFA. Peptides were separated during a biphasic ACN gradient from two nanoflow UPLC pumps (flow rate of 250 nl/min) on a 25 cm analytical column (PepMap RSLC, 50cm × 75 µm ID EASY-spray column, packed with 2 µm C18 beads). Solvents A and B were 0.1% FA (vol/vol) in water and 100% ACN respectively. The gradient composition was 5%B during trapping (5min) followed by 5–7%B over 0.5 min, 8–22%B for the next 80 min, 22–28%B over 10 min, and 35–80%B over 10 min. Elution of very hydrophobic peptides and conditioning of the column were performed for 15 min isocratic elution with 90%B and 20 min isocratic conditioning with 5%B. The eluting peptides from the LC-column were ionized in the electrospray and analyzed by the Orbitrap Eclipse. The mass spectrometer was operated in the DDA-mode (data-dependent-acquisition) to automatically switch between full scan MS and MS/MS acquisition. Instrument control was through Tune 2.7.0 and Xcalibur 4.4.16.14. Survey full scan MS spectra (from m/z 375 to 1500) were acquired in the Orbitrap with resolution R = 120.000 at m/z 200 (after accumulation to a target value of 4e5 in the C-trap, ion accumulation time was set to auto. FAIMS was enabled using two compensation voltages (CVs), − 45V and − 65V respectively. During each CV, the mass spectrometer was operated in the DDA-mode to automatically switch between full scan MS and MS/MS acquisition. The cycle time was maintained at 0.9s/CV. The most intense eluting peptides with charge states 2–6 were sequentially isolated to a target value (AGC) of 2e5 and a maximum IT of 120 ms in the C-trap, and isolation width maintained at 0.7 m/z, before fragmentation in the HCD (Higher-Energy Collision Dissociation) cell during the 1.5 s cycle time. Fragmentation was performed with normalized collision energy (NCE) of 30%, and fragments were detected in the Orbitrap at a resolution of 30,000 at m/z 200, with the first mass fixed at m/z 110. The spray and ion-source parameters were as follows. Ion spray voltage = 1900V, no sheath and auxiliary gas flow, and capillary temperature = 275 °C.

### Quantification of mitochondria morphology

Tubular cells’ mitochondria numbers and morphology were analyzed as previously reported [28]. Briefly, n = 16 (8/genotype, 4 male, 4 females) samples were used. Proximal tubule mitochondria (WT n = 1973 mitochondria, MU n = 945 mitochondria) and distal tubule mitochondria (WT n = 1697 mitochondria, MU n = 1260 mitochondria) were photographed at × 12,000 magnification.

Mitochondrial shape descriptors and size measurements were obtained using Image J (version 1.53r, National Institutes of Health, Bethesda, MD) by manually tracing only discernible outlines of tubular mitochondria on TEM micrographs.

### Morphological analyses of mitochondrial cristae

The same samples used for the mitochondria quantification were used for morphological analysis, following published guidelines [29]. Renal tubular cells were photographed at × 50,000 magnification for cristae morphology. Only clear mitochondria were selected for assessment. For proximal tubules, a total number of 143, and 192 mitochondria from WT and MU fish, respectively, was used. For distal tubules, 89 and 156 mitochondria from WT and MU fish, respectively, were used for the analysis. In addition, we evaluated individual mitochondria based on an adapted scoring scheme to assign cristae scores and evaluate cristae abundance and form [30]. Five-grade scoring system (from 0 the lowest cristae quality to 4 the highest cristae quality) was assigned to mitochondria based on cristae number and appearance.

### Immunohistochemistry

Kidney samples from adult ZF (90 + dpf) were used (n = 12, 6/genotype, 3 males, 3 females). IHC was performed as previously described [31] with slight modifications for each antibody. Antibodies used were Sod2 (RRID:AB_11174816) (1:50; without retrieval) and CD63 (RRID:AB_2800495) (1:800; retrieval pH9) from Abcam. Sections were incubated with primary antibodies for one hour at room temperature. For negative controls, the primary antibody was omitted. Slides were scanned with ScanScope XT^®^ (Aperio) at × 40 resulting in a resolution of 0.25 µm per Pixel. Digital slides were viewed in ImageScope 12.

After adjusting the default parameters to DAB staining, immunohistochemical positivity for each antibody was quantified using the color deconvolution algorithm version 9.1 (Aperio, CA, USA). The total percentage of positive pixels was used as a visualization parameter.

### Total antioxidants activity determination

Total antioxidant capacity determination was performed in 9 months old 8 WT and 8 MU ZF. After euthanasia, the body was dissected, and kidneys were extracted under cold 1X PBS (Life Technologies, Cat. No. AM9625), washed in cold 1X PBS, and snap-frozen in liquid nitrogen immediately. Total antioxidant activity was measured using Abcam kit ab65329 as per manufacturer instructions. SpectraMax Spectrophotometer (Molecular Devices) was used for optical density reading at 570 nm. The results were normalized by tissue weight.

### Experimental design and statistical rationale

For the proteome data analysis, we collected kidneys from 16 WT and 16 *gla*^*−/−*^ MU that were pooled for a final WT n = 8 and *gla*^*−/−*^ MU n = 8. Proteome Discoverer 2.5 (Thermo Fisher) was used to search the raw files from the Orbitrap Exploris. Using the built-in Sequest search engine with the FASTA file version from *Danio rerio* with 43644 entries_ 28102021. Normalized data from Proteome Discoverer 2.5 were transferred to Perseus version 1.5.5.3 [32] for statistical analysis. Significance was assessed using q-values combining FDR and Welch’s T-test, and a q-value ≤ 0.05 was considered significant. SPSS (IBM SPSS Statistics v.29) was used for general statistics. GO relevant terms and Kyoto Encyclopedia of Genes and Genomes (KEGG) enrichment analysis were retrieved from ShinyGO (http://bioinformatics.sdstate.edu/go/) [33–35]. GO terms with FDR ≤ 0.05 were considered significantly enriched.

For the assessment of mitochondria, cristae, and IHC (percent total positive pixels), statistical analysis was performed using GraphPad Prism V 9.2.0. Values are presented as violin plots (median/interquartile ranges) or as mean with an upper 95 confidence interval (CI). The Mann–Whitney test was used to assess statistical significance. Differences were considered significant with p-values ≤ 0.05.

## Results

### Proteomic profile of α-GAL mutant zebrafish reveals alterations independent of Gb3 accumulation

**Fig. 1:**
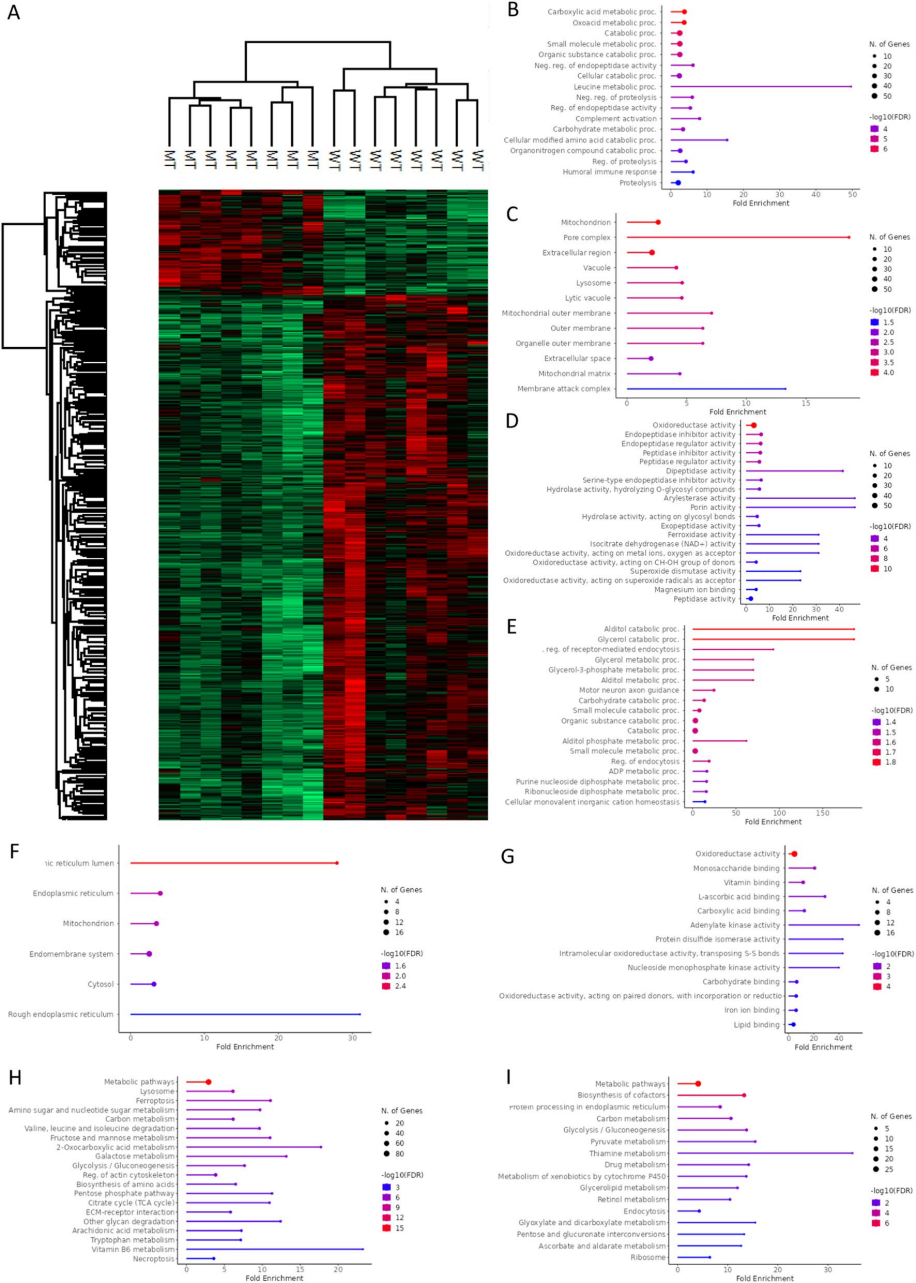
Proteomic comparison of kidneys from wild-type (WT; n = 8) and mutant (MU; n = 8) zebrafish. Gene ontology (GO) enrichment analysis and KEGG analysis. **A** Hierarchical clustering for differentially expressed proteins between WT and MT. Downregulated proteins in MU compared to WT in **B** GO Biological Process analysis; **C** GO Cellular component analysis; **D** GO Molecular function analysis. Upregulated proteins in MU compared with WT in **E** GO Biological Process analysis; **F** GO Cellular component analysis; **G** GO Molecular function analysis. **H** KEGG pathways related to downregulated proteins in MU compared to WT. **I** KEGG terms associated with upregulated proteins in MU vs. WT. Enrichments are reported as false discovery rates (FDR) ≤ 0.05. The twenty most enriched pathways are represented

To assess kidney proteome alterations, we comparatively analyzed renal tissues from wild-type (WT, n = 8) and *gla*^*−/−*^ mutant (MU, n = 8) ZF. A total of 7110 proteins were identified by mass spectrometry, and 6770 of them, showing high false discovery rate (FDR) confidence, were included in subsequent statistical analysis. Identified proteins and statistics are included in Additional file 1. We observed that 639 proteins were differentially expressed (Welch T-test ≤ 0.05 with a q-value ≤ 0.05). In particular, 527 proteins were downregulated and 112 upregulated in *gla*^*−/−*^ MU vs. WT renal samples (Fig. 1A).

Gene ontology (GO) analysis showed that proteins downregulated in *gla*^*−/−*^ MU vs. WT ZF were mainly involved in the biological processes (BP) of carbon metabolism, including carboxylic acid and carbohydrate metabolism, or of small molecule metabolism (Fig. 1B). As for cellular components (CC), mitochondria, extracellular region, pore complex and lysosomes were the most cited GO terms (Fig. 1C). Oxidoreductase activity, Endopeptidase inhibitor activity, Peptidase inhibitor, isocitrate dehydrogenase (NAD^+^), superoxide dismutase, and glutathione hydrolase activities were highly represented among molecular function (MF) terms (Fig. 1D). In upregulated proteins in MT vs. WT ZF, GO terms included alditol and glycerol catabolic processes in BP (Fig. 1E), endoplasmic reticulum and mitochondrion in CC (Fig. 1F), and oxidoreductase activity, monosaccharide, and vitamin binding in MF (Fig. 1G).

Kyoto Encyclopedia of Genes and Genomes (KEGG) pathways analysis revealed a downregulation of lysosome, mitochondrion, and energy-related pathways in *gla*^*−/−*^ MU ZF. They included citrate cycle, carbon, 2-oxocarboxylic acid, galactose, vitamin B6, amino acid-sugar and nucleotide-sugar metabolisms. Inflammation-related processes, such as ferroptosis and necroptosis were also affected (Fig. 1H).

Instead, KEGG terms associated with upregulated proteins in MU vs. WT specimens included metabolic pathways, biosynthesis of cofactors and drug metabolism (Fig. 1I).

These proteomics results follow our transcriptomics data from the same model [36] where mitochondrion and energy-related pathways, lysosomes and carbon metabolism were also down-represented in the *gla*^*−/−*^ MU compared to the WT ZF.

### Mutation in *gla* affects lysosome-related proteins in a Gb3-independent manner

FD is a lysosomal storage disorder, and the disruption of lysosomal protein trafficking/sorting as well as of the autophagy process are thought to be influenced by Gb3 accumulation [37]. However, most importantly, *gla* gene mutation in Gb3 synthase deficient ZF was also associated with lysosome dysregulation. Indeed, KEGG analysis revealed that the expression of several lysosome-related proteins was statistically lower in *gla*^*−/−*^ MU than in WT ZF renal tissues. Besides α-Gal, these proteins included enzymes such as Cathepsins (Ctsba, Ctsz, Ctsla, Ctsf), Glucosylceramidase (Gba), Neuraminidase 1 (Neu1), Deoxyribonuclease II (dnaseII), Aspartylglucosaminidase (Aga), palmitoyl-protein thioesterase 1 (Ppt1/Cln1), Hexosaminidase A (Hexa), and lysosomal membrane proteins including Lysosomal-associated membrane protein 1a (Lamp), Tetraspanin (Cd63/Limp1) and Intracellular cholesterol transporter 1 (Npc). Moreover, proteins and receptors associated with the transport of synthesized lysosomal enzymes, such as Mannose-6-phosphate receptor (Mpr), and Adaptor-related protein complex 1 (Ap-1) and 3 (Ap-3) were also significantly downregulated (Additional file 1).

### Mutation in *gla gene* perturbs mitochondrial morphology

**Fig. 2:**
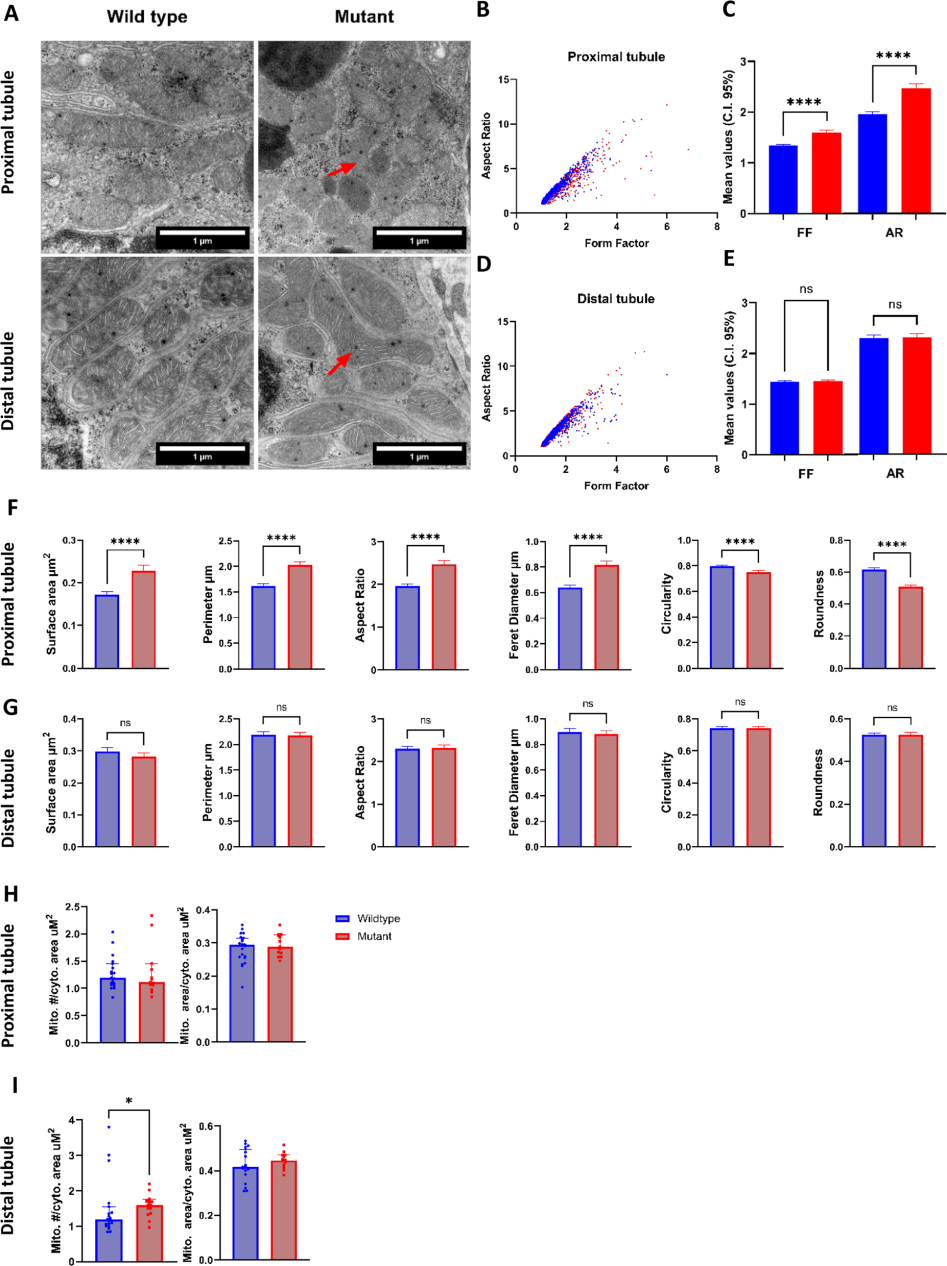
Analysis of morphological parameters in wild-type and GLA-mutant mitochondria in cells from proximal and distal renal tubules. **A** Electron microscopy showing representative micrographs of mitochondria (red arrows) in cells from wild-type and mutant proximal and distal tubules; **B** Mitochondrial form factor and aspect ratio plotted against each other in proximal tubule; **C** Statistical comparison using mean with 95% confidence intervals (CI) for form factor and aspect ratios in mitochondria from proximal tubule cells; **D** Mitochondrial form factor and aspect ratio plotted against each other in distal tubule; **E** Statistical comparison using mean and 95% confidence intervals (CI) for form factor and aspect ratios in mitochondria from distal tubule cells; **F**, **G** Mitochondrial morphological parameters comparison in proximal tubules and distal tubules; **H**, **I** Mitochondrial volume and density determination in proximal and distal tubules. Values are represented as mean with upper 95% CI. Data were analyzed using Mann–Whitney test. *p-value ≤ 0.05; ****p-value ≤ 0.00001; ns = non-significant

Mitochondrial morphology is intrinsically associated with function. Since we had observed differences in the proteome and oxidative stress levels in mitochondrial-related proteins, we used transmission electron microscopy to evaluate mitochondrial morphology. Mitochondria were individually traced from transmission electron micrographs (Fig. 2A).

This analysis documented significant alterations of mitochondrial morphology in renal tissues from *gla*^*−/−*^ MU, compared to WT ZF. Most importantly, altered morphology was almost exclusively observed in proximal tubules (Fig. 2B and C), whereas distal tubules appeared to be largely unaffected as shown by the mean values of the form factor and aspect ratio (Fig. 2D and E). In particular, in mitochondria from proximal tubule cells derived from *gla*^*−/−*^ MU ZF, shape parameters [28] were significantly distorted, compared with WT specimens (Fig. 2F and G).

However, mitochondrial volume and density in cells from proximal tubules were similar in *gla*^*−/−*^ MU and WT samples (Fig. 2H), whereas in distal tubules mitochondrial density was higher in *gla*^*−/−*^ MU, compared to WT ZF (Fig. 2I).

**Fig. 3:**
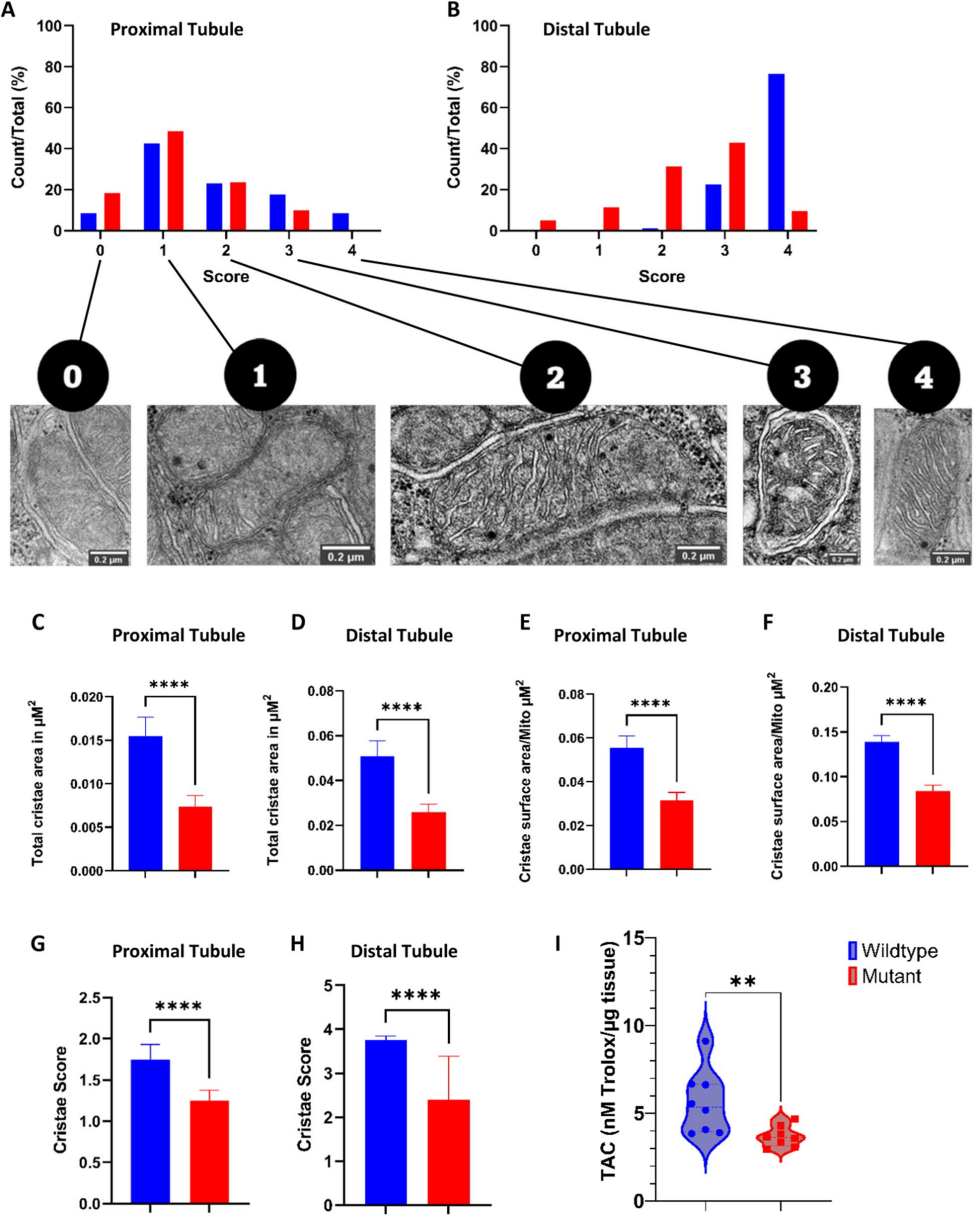
Mitochondrial cristae morphology and area determination in proximal and distal tubules performed using TEM images and total antioxidant determination (TAC) in wild-type and GLA-mutant Zebrafish. **A**, **B** Individual score grades across the whole mitochondrial population analyzed in cells from renal proximal and distal tubules; **C**, **D** Total cristae area in cells from proximal and distal tubules; **E**, **F** Cristae volume in proximal and distal renal tubules; **G**, **H** Cumulative cristae mean scores for proximal and distal renal tubules, **I** TAC levels in adult (9-months-old) zebrafish kidney tissue lysates. For mitochondria cristae morphology quantification data represented as mean with upper 95% confidence interval. For TAC values are represented as violin plot representing the median and IQ ranges. Data were analyzed using Mann–Whitney test; ****p-value ≤ 0.00001; **p-value ≤ 0.001

Cristae represent the main functional component of mitochondria, and their activity is dependent on the surface area where chemical reactions do occur. Therefore, we quantified cristae alterations in proximal and distal tubular cells from both *gla*^*−/−*^ MU and WT tissues obtained from adult ZF (Fig. 3A and B). Disruption of mitochondrial cristae morphology and volume per mitochondria, reduction of average cristae surface area, and cristae volume/density were detected in cells from both proximal and distal tubules derived from renal tissues of *gla*^*−/−*^ MU fish (Fig. 3C–F). Accordingly, cristae scores [30] revealed a reduction of healthy mitochondria numbers in cells from *gla*^*−/−*^ MU proximal and distal tubule relative to WT (Fig. 3G and H), indicating perturbations in the oxidative phosphorylation system, which relates with impairing cellular metabolism and growth.

### Oxidative stress is increased in the *gla*^*−/−*^ mutant zebrafish

Gb3-dependent dysregulated autophagy and autophagic flux are known to induce severe oxidative stress in FD [38, 39], and other metabolic disorders [40–43], by disrupting mitochondrial function. Indeed, our proteomics results revealed that several mitochondrial-related pathways were markedly affected even without the presence of Gb3.

To assess oxidative stress in our model, total antioxidant capacity (TAC) assay was performed, finding that TAC was significantly lower in renal tissue lysates from adult *gla*^*−/−*^ MU as compared to WT ZF (Fig. 3I).

### Protein validation by immunohistochemistry

**Fig. 4:**
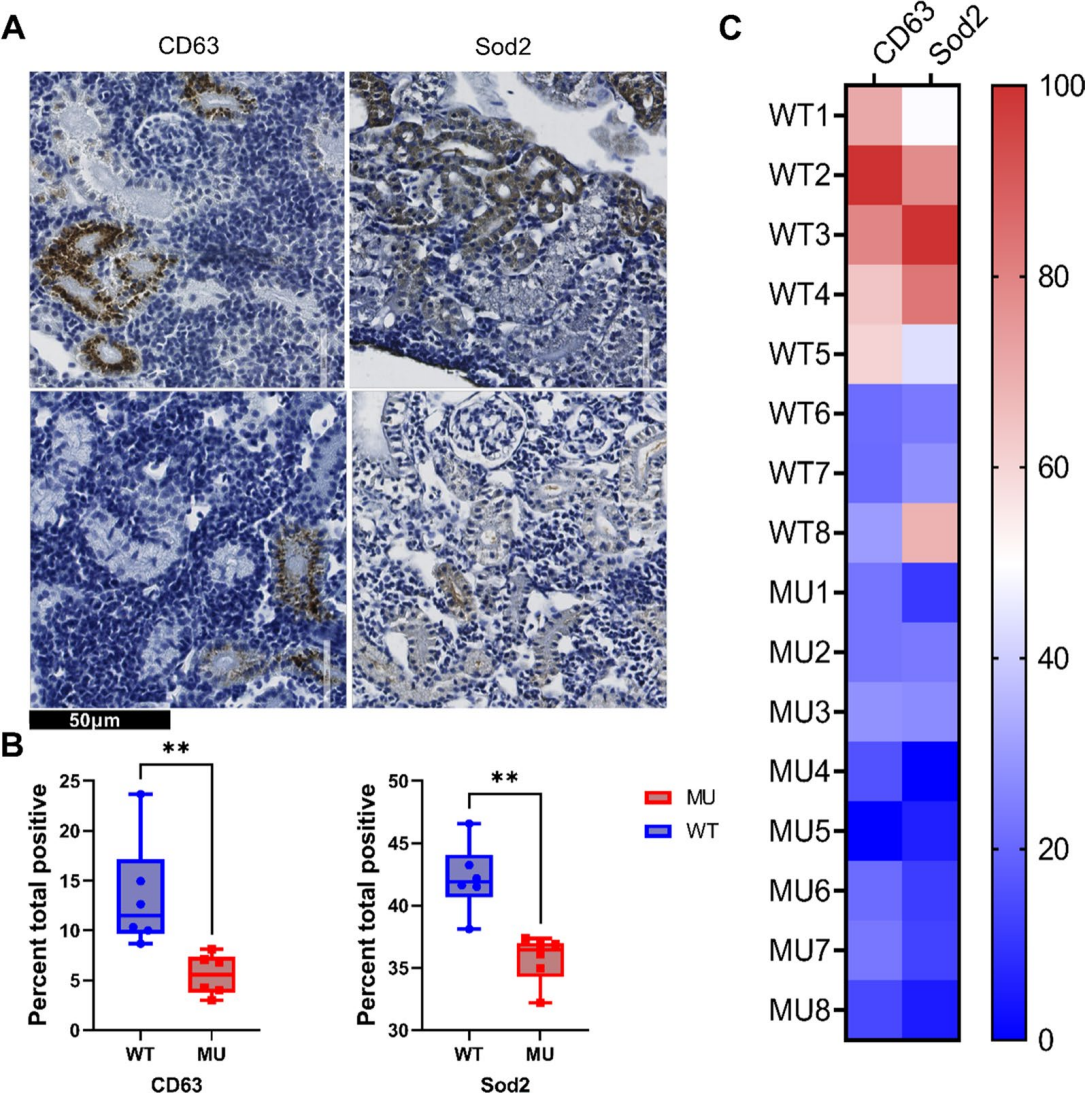
Immunohistochemical analysis of the expression of selected proteins in wild-type and mutant zebrafish renal tissues. **A** Representative image of IHC analysis targeting mitochondrial marker Sod2 and lysosomal marker Cd63 in kidneys from wild-type and mutant ZF renal tissues; **B** Quantification of immunohistochemical staining of sections from wild-type and mutant ZF kidneys. Signal intensity is significantly higher in wild-type than in mutant specimens for CD63 and Sod2; **C** Heatmap showing protein expression of CD63) and Sod2 in mutant and wild-type kidney tissues, normalized as percent of positive pixels. Data analysis using Mann–Whitney test; **p-value ≤ 0.001

Immunohistochemistry was used to validate differential protein expression of mitochondrial, lysosomal, and adhesion junction proteins which were selected based on the extent of their dysregulation and the availability of commercially validated zebrafish-specific antibodies. Sod2 (mitochondria) and Cd63/Limp (lysosome) were studied in detail (Fig. 4A). In accordance with proteomic data, a semiquantitative immunohistochemical analysis demonstrated reduced average signals in *gla*^*−/−*^ MU, compared to their WT counterparts (Fig. 4B), as measured by the percentage total positive pixels. Average protein expression represented as the percent of positive pixels in each sample is shown in Fig. 4C.

## Discussion

Our findings unravel several important alterations of cell functions mirroring those observed in FD patients [39, 44, 45]. However, the spectrum of observed changes occurring in *gla*^*−/−*^ MU ZF in the absence of Gb3 accumulation adds important novel insight to the disease mechanisms involved in FD.

Our GO term analysis revealed that lysosome and mitochondrial-related proteins are downregulated in *gla*^*−/−*^ MU vs. WT renal tissues, whereas energy-related pathways including carbon, glycolysis and galactose metabolisms are disturbed in the *gla*^*−/−*^ MU. This is consistent with disrupted autophagy and impaired mitophagy processes that are critical for cellular homeostasis. These alterations have been observed in FD patients and other animal models with Gb3 accumulation, being suggested that FD is associated with impaired mitochondrial function, including reduced mitochondrial respiration and ATP production. This can lead to decreased energy production, potentially contributing to the fatigue and exercise intolerance observed in Fabry disease patients [46, 47]. Our data suggest that these changes can occur in the absence of Gb3 accumulation, pointing to additional pathways that lead to an FD-related phenotype.

Indeed, not only were different lysosomal enzymes, including Gba, Neu1, and Aga, underrepresented in MU ZF, but lysosomal membrane proteins Lamp and Limp/Cd63 and transport proteins Ap-1 and Ap-3 were also underrepresented, consistent with disrupted autophagy in this FD model.

The inability of the lysosome to integrate into the mitophagosome might lead to an impaired downstream mitophagy process, thus explaining the elevated oxidative stress and the altered mitochondrial morphology [39, 40, 43, 45, 48] observed in *gla*^*−/−*^ MU ZF renal tissues.

An interesting case worth mentioning is Limp/CD63. CD63 is known to be involved in the lysosome sorting procedure [49, 50]. In our data, Cd63 is lower in the *gla*^*−*/−^ MU ZF, however, in a α-GAL-deficient podocyte cell line model for FB, Cd63 protein expression remained unaltered [51]. A possible reason for the discrepancy between the latter study and ours is that in the absence of Gb3 accumulation, additional pathways leading to an FD-related phenotype may be triggered. Also, the transit of Limp-1/Cd63 from the trans-Golgi network to lysosomes requires the AP-3 adaptor complex [52], which was also downregulated in our dataset. Nevertheless, CD63 inactivation in mice causes polyuria and decreased urine osmolality, indicating that lysosomes contribute to renal homeostasis [53]. Lysosome-integrated membrane proteins were previously shown to play crucial roles in lysosomal-related diseases [54]. Since CD63 is detectable in the urine of chronic kidney disease (CKD) model rats [55] as well as in human urine [56–58], it might be considered a novel, non-invasive potential biomarker for FD.

For validation of our proteomic data supporting mitochondrial dysfunction at the functional and morphological level, we used an oxidative stress assay and transmission electron microscopy. At the functional level, we observed an altered antioxidant activity in MU, compared to WT renal tissues. Similar findings have previously been observed in FD patients [38], *GLA*-KO human iPSC kidney organoids [59] and FD patients-derived renal tubular epithelial cell lines [39]. Therefore, oxidative stress has emerged as a possible indicator of FD in conjunction with Gb3 buildup [21]. Furthermore, oxidative stress was also observed in FD patients with normal lysoGb3 levels [60]. Our data indicate that oxidative stress can be initiated and maintained in the absence of Gb3.

Further analysis of our proteomics data highlighted the disruption of Sod2 activity, which was confirmed through immunohistochemical staining. Previous studies have associated reduced Sod2 activity with increased oxidative stress in zebrafish [61]. Consistent with these findings, our data revealed down-regulation of Sod2 in *gla*^*−/−*^ MU ZF. Similar alterations in Sod2 expression have been observed in pluripotent stem cells derived from the peripheral blood of FD patients [62], where Sod2 downregulation was attributed to Gb3 accumulation. However, our results suggest that Sod2 disruption can occur independently of Gb3, indicating the need for further investigation to unravel the complex regulation of Sod2 under different pathological triggers.

Mitochondrial physiology is closely linked to morphology, as evidenced by previous studies on various lysosomal storage disorders [41, 42, 63]. Mitochondrial abnormalities have been observed in human renal tubular epithelial cell lines derived from FD patients and in an FD-mouse model [64]. In our investigation, mitochondrial shape was abnormal in proximal tubule cells from *gla*^*−/−*^ MU, compared to WT ZF, although the proportion of cytoplasm covered by mitochondria remained unmodified. Our findings differ from those of Nagano et al., [65], who reported that Gb3 increase was proportional to the reduction of the percentage area of mitochondria in the cytoplasm in FD patients. This discrepancy could be due to a lack of Gb3 buildup in our model. Notably, mitochondrial morphological alterations were not detectable in the FD-mouse model [66], where, however, no clinical symptoms of renal disease were observed at 10 weeks of age. Interestingly, in the *gla*^*−/−*^ MU ZF, we observed disrupted cristae morphology and volume, which are critical for the chemical reactions occurring on the inner membrane of mitochondria. These alterations were independent of the external mitochondrial morphology. Consistent with our findings, swollen mitochondria and disorganized cristae structures have been described in human renal tubular epithelial cells derived from urine of FD patients [39]. Based on these data, we propose that while external morphology is only affected in distal tubules and not in proximal tubules, the smaller cristae surface area indicates reduced mitochondrial activity. In addition to the presented proteomic data, our previous transcriptomic findings using this ZF model also revealed significant dysfunctions in mitochondrial and energy-related processes [36] consistent with the proteome findings. Interestingly, we did not detect the presence of lysosome-related RNAs in our transcriptome dataset. It is essential to emphasize that while gene expression data offer valuable insights into differential gene expression following experimental stimuli or describe basal gene expression during biological processes, transcriptome analysis represents a relatively indirect approach to studying organelle function. The complexities arising from post-transcriptional regulation and RNA dynamics can significantly impact the detectability of RNA transcripts. RNA transcription may be subject to disruption, and RNA molecules may undergo rapid degradation or recycling following translation, rendering them transient and challenging to detect through transcriptome analysis.

The limitations of this study should be acknowledged. While the results suggest promising avenues for drug screening and the identification of clinical targets, it is essential to acknowledge the need for further research and validation to support the clinical translation of these findings. Animal models, in vitro studies, and, ultimately, clinical trials would be required to confirm the functional significance of the identified Gb3-independent alterations and to assess the safety and efficacy of any potential therapeutic interventions targeting these pathways. Also, limitations due to the nature of FD should be acknowledged. Indeed, pathophysiological gender disparities do represent a challenge [67], because in humans the *GLA* gene is located on the X chromosome, but in ZF it is located on an autosomal chromosome. Hence, pathophysiological differences between male and female zebrafish cannot be reliably addressed [68–70].

## Conclusions

Our findings demonstrated significant alterations in cell functions mirroring those observed in FD patients and provide novel insights into Gb3-independent mechanisms underlying FD progression. The observed disruptions in lysosome-related proteins, energy metabolism pathways, and mitochondrial structure and function indicate the involvement of alternative pathways in FD pathogenesis. Thus, although Gb3/lysoGb3 accumulation might contribute to their progression, other mechanisms are involved in the initiation and maintenance of these processes in FD. Our findings unravel Gb3-independent mechanism in FD progression and might pave the way toward the development of innovative diagnostic, monitoring and, potentially, therapeutic procedures beyond mechanisms related to Gb3 accumulation.

### Supplementary Information

**Additional file 1.** Proteins identified by mass spectrometry including statistics.

## Data Availability

The raw files and search results are available in the ProteomeXchange Consortium (http://proteomecentral.proteomexchange.org/cgi/GetDataset) via the PRIDE partner repository [71] with the project accession number: PXD035409.

## Abbreviations

FD: Fabry disease
Gla: Galactosidase alpha
Gb3: Globotriaosylceramide
ZF: Zebrafish
A4GALT: Alpha 1,4-Galactosyltransferase
lysoGb3: Globotriaosylsphingosine
ERT: Enzyme replacement therapy
ESRD: End-stage renal disease
AB/TU: AB/Tübingen strain
MU: Mutant
WT: Wild-type
DTT: Dithiothreitol
IAA: Iodoacetic acid
AmBic: Ammonium bicarbonate
CaCl2: Calcium chloride
TMT: Tandem Mass Tag
ACN: Acetonitrile
FA: Formic acid
GO: Gene ontology
BP: Biological processes
CC: Cellular components
MF: Molecular function
KEGG: Kyoto Encyclopedia of Genes and Genomes
TAC: Total antioxidant capacity
IHC: Immunohistochemistry
α-GAL: Alpha-galactosidase
Cts: Cathepsin
Gba: Glucosylceramidase
Neu1: Neuraminidase 1
dnaseII: Deoxyribonuclease II
Aga: Aspartylglucosaminidase
Ppt1/Cln1: Palmitoyl-protein thioesterase 1
Hexa: Hexosaminidase
Lamp: Lysosome-associated membrane protein 1a
Cd63/Limp1: Tetraspanin
Npc: Intracellular cholesterol transporter 1
Mpr: Mannose-6-phosphate receptor
Ap-1: Adaptor-related protein complex 1
AP3: Adaptor-related protein complex 3
Sod2: Superoxide dismutase 2
